# Microbial Growth and Biogenic Amine Production in a Balkan-Style Fresh Sausage during Refrigerated Storage under a CO_2_-Containing Anaerobic Atmosphere: Effect of the Addition of *Zataria multiflora* Essential Oil and Hops Extract

**DOI:** 10.3390/antibiotics8040227

**Published:** 2019-11-15

**Authors:** Diego E. Carballo, Javier Mateo, Sonia Andrés, Francisco Javier Giráldez, Emiliano J. Quinto, Ali Khanjari, Sabina Operta, Irma Caro

**Affiliations:** 1 Department of Hygiene and Food Technology, Faculty of Veterinary Medicine, University of León, 24071 León, Spain; diegocarballo2@hotmail.com (D.E.C.); jmato@unileon.es (J.M.); 2 Instituto de Ganadería de Montaña, CSIC-Universidad de León, Finca Marzanas s/n, Grulleros, 24346 León, Spain; sonia.andres@eae.csic.es (S.A.); j.giraldez@eae.csic.es (F.J.G.); 3 Department of Nutrition and Food Science, Faculty of Medicine, University of Valladolid, 47005 Valladolid, Spain; irma.caro@uva.es; 4 Department of Food Hygiene, Faculty of Veterinary Medicine, University of Tehran, P.O. Box 14155-6453, Tehran, Iran; khanjari@ut.ac.ir; 5 Institute of Food Science and Technology, Faculty of Agricultural and Food Science, University of Sarajevo, 71000 Sarajevo, Bosnia and Herzegovina; s.operta@ppf.unsa.ba

**Keywords:** lamb sausage, lactic acid bacteria, shelf-life, natural antimicrobials, meat preparations, modified atmosphere packaging

## Abstract

Fresh sausages are highly perishable, and the preservatives allowed in these types of meat preparations are limited. Balkan-style fresh sausages were prepared in triplicate without antimicrobials (Control), with an aqueous hops extract (30 mL/kg), with *Zataria multiflora* Boiss essential oil (1 mL/kg), or a combination of both (15 and 0.5 mL/kg, respectively), and refrigerator-stored under a 20% CO_2_ and 80% N_2_ atmosphere. The spoilage microbial growth, i.e., lactic acid bacteria (LAB), *Brochothrix thermosphacta*, *Enterobacteriaceae*, *Micrococcaceae*, molds and yeasts, the pH value, and the production of biogenic amines in the sausages were monitored weekly and compared with a control sausage during a 35-day storage period. Furthermore, 349 colonies of presumptive LAB (isolated from the De Mann, Rogose-Sharpe agar plates) were identified using a MALDI-TOF-based method. Growth levels to ≈ 9 Log colony forming units (CFU) per g were reached by LAB, with a predominance of *Lactobacillus sakei*. *Enterobacteriaceae* and *B. thermosphacta* also showed significant growth (up to 6 Log CFU/g). Biogenic amine levels increased, and tyramine values overcame 250 mg/kg. The study could not demonstrate a significant effect of antimicrobial source treatments in any of the characteristics studied, and thus, the shelf-life of sausages.

## 1. Introduction

Fresh sausages are produced with comminuted meat, salt, species, and condiments and a limited number of allowed additives. Their formulation, preparation, and dimensions strongly depend on local preparation. Fresh sausages must be refrigerator-stored and cooked before consumption. They are considered to be highly perishable, with pH values >5.5 and water activity (a_w_) ≥0.97 [1]. To retard microbial growth, fresh sausages are commonly stored at low temperatures under anaerobic CO_2_-containing modified atmosphere packaging (MAP). The spoilage microbiota of fresh sausages on these conditions consists of facultative anaerobic microorganisms such as lactic acid bacteria (LAB), *Brochothrix thermosphacta*, and *Enterobacteriaceae*, with LAB being observed as the predominant group [2,3]. The shelf-life of fresh sausages refrigerator-stored under anaerobiosis depends on the hygienic quality of raw materials, pH, a_w_, storage temperature, atmosphere, etc. [4]. Some authors, based on the appearance of off-odors and discoloration, have found a shelf-life for these sausages slightly longer than 10 days [3] and others of more than 20 days [5,6,7].

During refrigerated storage of fresh sausages under vacuum or anaerobic MAP, and most probably due to the growth of LAB and *Enterobacteriaceae*, a significant production of biogenic amines (BA) such as tyramine, putrescine, and cadaverine occurs [4,8]. In a previous study [2], levels of tyramine higher than 100 mg/kg were found in a Mexican fresh sausage stored in anaerobic MAP for more than two weeks, which represents a health risk and corroborates the need to control the production of BA in fresh sausages.

A current approach to extend the shelf-life of fresh sausages is the use of natural antimicrobials [9]. Hops, the strobiles (female flowers) of the *Humulus lupulus* L. plant, which are commonly used in brewery and have found application in other foods [10], appear to be a potentially suitable ingredient for this purpose. Hops contains antimicrobial compounds, such as prenylated acylphloroglucinols, bitter acids or xanthohumol, among others, which have been probed to inhibit Gram-positive bacteria [11,12]. The Food Safety and Inspection Service from the USA has approved the use of hops *α*-acids as antimicrobials for cooked meat and casings [13]. Moreover, Kramer et al. [11] found hops extract to inhibit total aerobic microbial growth in marinated pork. However, the effect of hops in fresh sausages packaged under anaerobic conditions seems to have been rarely studied. Hops could interfere in the growth of Gram-positive spoilage microorganisms such as LAB or *Brochothrix thermosphacta*, thus extending the sausage shelf-life.

Plant-derived essential oils (EO) obtained from aromatic and medical plant materials have proved wide antimicrobial spectra against bacteria, yeasts, and molds [14]. Nonetheless, among bacteria, the Gram-positive are more susceptible than the Gram-negative [15]. The effectiveness of EO at levels up to 2% in extending the lag phase or reducing the final population of spoilage microbiota in minced meat and meat products during refrigerated storage has been reported [16,17]. EO has been claimed to be one of the best alternatives to synthetic preservatives in meat and meat products [17]. However, the use of EO in meat as well as in other foods as natural preservatives present relevant limitations regarding deleterious effects in sensory quality due to their strong flavor, loss of antimicrobial activity due to interactions with food components, and regulatory or safety issues [18]. In this context, the use of EO combined with other synergistic or complementary natural antimicrobials has been suggested as a viable approach to using lower amounts of EO, thus not affecting the sensory acceptation, while achieving a significant antimicrobial effect.

Among the EO, that obtained from *Zataria multiflora* Boiss (ZM), which contains carvacrol and thymol as its main components, has shown a significant antimicrobial effect, this effect being greater on Gram-negative bacteria [19,20,21]. *Zataria multiflora* Boiss, belonging to the *Laminaceae* family, is cultivated in warm parts of the Middle East, where it is popularly used in traditional medicine and as food flavoring and preservative [22]. Regarding processed meat, it has been reported that the addition of ZM’s EO at levels up to 0.1% reduced the growth of total viable microbiota, *Pseudomonas* spp., and LAB in buffalo burgers during aerobic storage [23,24]. Moreover, a chitosan film containing this EO (5–10 g/kg) also reduced the counts of total viable microbiota at the surface of mortadella-type slices packaged in oxygen permeable polyethylene bags during refrigerated storage [25]. The above-mentioned studies were carried out using aerobic storage; however, no study has been found in the literature addressing the antimicrobial effect of ZM’s EO on meat or meat products stored under anaerobic MAP.

This study has aimed to evaluate the growth of spoilage microorganisms and BA production in a typical fresh sausage during refrigerated storage under CO_2_ plus N_2_ MAP, and to assess the effect of two natural antimicrobials: hops and ZM’s EO, alone or combined. The study focused on LAB population considering them as the predominant spoilage microorganisms in fresh sausages packaged under this type of atmosphere and responsible for the BA formation.

## 2. Results and Discussion

### 2.1. Water Activity, pH, Microbial Contents, and Biogenic Amine Production

The mean (standard deviation) a_w_ and pH values of the sausages from the three batches at day 0 were 0.987 (±0.004) and 6.01 (±0.02), respectively. During storage, the pH values decreased steadily (*P* < 0.05) from day 7 to day 28 for all the treatments, with the effect of either treatment or treatment x storage time interaction being non-significant. The mean values of pH in sausages (the four treatments) at days 7, 14, 28, and 35 were 6.05 (±0.01), 5.89 (±0.01), 5.66 (±0.02) and 5.61 (±0.03), respectively (data not shown in tables for brevity).

**Figure 1:**
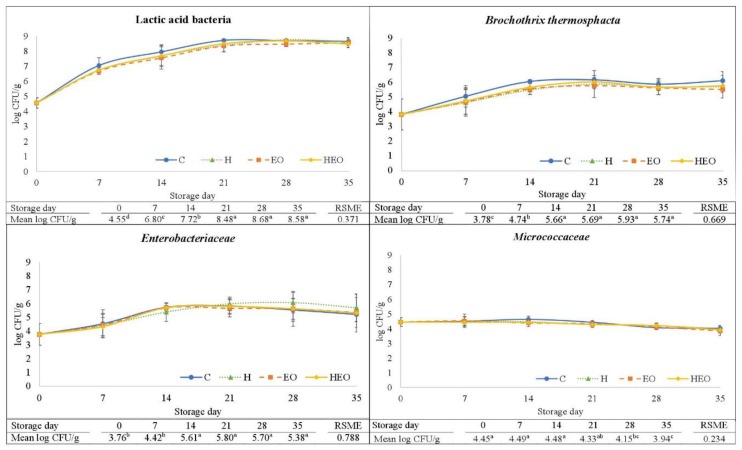
Effect of the addition of different natural antimicrobial sources on lactic acid bacteria: *Brochothrix thermosphacta*, *Enterobacteriaceae*, and *Micrococcaceae* counts (mean values, *n* = 3, and standard deviation, vertical bars) in fresh lamb sausages packaged under modified atmosphere (80% N_2_, 20% CO_2_) during refrigerated storage (4 °C). CFU: Colony forming units. RSME: Root square mean error. C: Control sausages; H: hops; EO essential oil (*Zataria multiflora* Boiss); HEO: hops and essential oil. ^abcd^: Total means (*n* = 12) with different superscripts time-related indicate statistical differences (Tukey test, *P* < 0.05).

As is shown in Figure 1, the counts of LAB, *B. thermosphacta*, *Enterobacteriaceae,* and *Micrococcaceae* were not significantly affected by antimicrobial treatment. The mean counts of the Gram-positive LAB and *B. thermosphacta* tended to be higher in control (C) sausages, although the *P* values from the analysis of variance (ANOVA) were not significant, i.e., 0.263 and 0.397, respectively (not shown in figures). ZM’s EO contains high amounts of antimicrobial molecules, i.e., thymol, carvacrol, *α*-terpinene, and a contrasted antimicrobial effect in in vitro experiments [19,21]. In this study, the lack of effect of ZM’s EO on microbial growth in the fresh sausages could be explained by a loss of inhibitory efficacy due to interactions between the antimicrobials and sausage matrix compounds such as fat or specific proteins, which could be influenced by the sausage pH and a_w_ [15,18].

In contrast with our results, the addition of different EO, i.e., bay leaf, cassia, clove, holy basil, lemon, thyme, or sage, to fresh sausages at levels between 0.01% and 0.25% has shown significant reducing effects on the growth of spoilage microflora during refrigerated storage of fresh sausages packaged under aerobic atmosphere [26,27,28,29]. Moreover, the ZM’s EO at levels up to 0.1% also significantly decreased the growth of total viable microbiota, *Pseudomonas* spp. and LAB in burgers during aerobic refrigerated storage [23,24]. Nevertheless, a clear difference between those studies and this one is that in the formers, the atmosphere was aerobic and in this study it was anaerobic. This suggests that the antimicrobial effect of EO in fresh comminuted meat products might be higher on the microbiota growing in fresh minced meat products with O_2_ than in that growing when the presence of O_2_ is restricted.

Hops extracts have been demonstrated to be useful as antimicrobials in casings, cooked ready-to-eat meat, and marinated meat products [11,13]. However, no study has been found investigating their antimicrobial effect in fresh comminuted meat products. In this study, hops extract given alone or combined with ZM’s EO did not reduce the growth of spoilage bacteria. Again, chemical interactions between the hops antimicrobials and the food matrix would be the reasons for the lack of significant antimicrobial activity. In order to achieve a positive antimicrobial effect due to the use of hops extracts in fresh sausages stored under anaerobiosis, it is suggested to use a higher amount of hops antimicrobials or reduce the sausage pH, due to the reported higher effect of hops in food matrix with pH close to 5 [11].

Regarding the changes on microbial growth (Figure 1), LAB became the dominant microbial group from day 7 onwards, reaching final values slightly higher than 8 Log colony forming units (CFU) per g at day 21, which can be considered as the onset of the stationary growth phase. Psychrotrophic LAB have been found to become the major microorganisms in fresh sausages refrigerator-stored under anaerobic CO_2_-containing MAP over the third week of storage [3,30], with the maximum LAB levels being comparable to those from this study. Lactic acid bacteria are considered as the principal spoilage-specific microorganisms in meat and fresh sausages stored under vacuum or CO_2_-containing anaerobic MAP [31]. Thus, the appearance of off-flavors, i.e., sour or putrid, in fresh pork sausage has been related to LAB counts over 7–8 Log CFU/g [5,32].

Both *B. thermosphacta* and *Enterobacteriaceae* showed similar growth patterns between them, i.e., starting with counts near to 4 Log CFU/g at day 0 and reaching the stationary phase at day 14 with counts of 5–6 Log CFU/g (Figure 1). In both cases, the growth phase was slower and shorter than that for LAB, which suggests a competitive effect of LAB, probably due to a higher ability of LAB for the consumption of limiting nutrients under the anaerobic MAP fresh sausage conditions [33].

Other studies also described how *B. thermosphacta* steadily increases its levels in fresh meats during refrigerated storage under vacuum and anaerobic MAP, becoming one of the dominant spoilage species and originating cheesy, buttery, or sour odors [34]. According to Samelis [35], the levels of *B. thermosphacta* associated to fresh meat spoilage are around 7 Log CFU/g.

The control of *Enterobacteriaceae* in fresh sausages seems to be desirable since levels of 4–5 Log CFU/g [36] have been associated with meat spoilage—counts higher than this level were overcome in this study at day 14. The growth pattern of *Enterobacteriaceae* in fresh sausages stored under CO_2_- and N_2_-containing MAP has shown variability among studies. In agreement with our results, Benson et al. [32] reported an exponential growth of *Enterobacteriaceae* during the first two weeks of storage of a fresh pork sausage, reaching counts around 6 Log CFU/g; however, Ruíz-Capillas and Jiménez Colmenero [3] found the levels of *Enterobacteriaceae* in fresh pork sausages to decrease after 10 days of refrigerated storage. These differences might be explained by variations among studies in spice mixtures, sausage pH, or bacterial communities and their competence.

*Micrococcaceae* counts were stable up to day 21 of storage and then decreased slightly until day 35 (Figure 1). A decrease after some weeks of storage has been described in other studies on fresh sausages during anaerobic refrigerated storage [37,38], and attributed to both pH decrease and low O_2_ and nutrients availability. No differences were found due either to treatment nor storage time in the molds and yeast counts (the mean values considering all the treatments and days were 2.99 ± 0.17 Log CFU/g; *n* = 24; data not shown in tables for brevity), which is probably due to their low growing ability under diminishing O_2_ levels [38].

Changes in BA production in sausages are shown in Table 1. The levels of BA were not affected by antimicrobial treatment except for spermine (*P* = 0.037), with slightly higher amounts in the hops extract and essential oil (HEO) sausages than in the C sausages. Mono and diamines in fresh sausages are presumably produced from microbial enzymatic decarboxylation of free amino acids. In ripened sausages, this is mainly carried out by LAB and *Enterococci*, this ability being strain-dependent [39]. *Enterobacteriaceae* and *B. thermosphacta* can also contribute to the production of BA in LAB-fermented meats, with the first being more active in cadaverine and putrescine formation and the latter in histamine and tyramine [40,41,42]. The lack of effect of treatment on BA in the fresh sausage is thus coherent with the absence of a significant effect on microbial growth. However, the levels of tryptamine, putrescine, and histamine were significantly different between the experimental batches (data not shown in tables). Thus, the levels of putrescine and histamine in the second batch were respectively more than 5 times and 20 times higher than in the other two batches, which would corroborate the dependence of BA production on microbial strains.

In contrast with our results, Lu et al. [43] reported a reduction in biogenic mono and diamine production in Chinese smoked sausages as a result of the addition of a mixture of essential oils (from cinnamon, cloves, ginger, and anise; 0.12% in total) and tea polyphenols (0.19%) to the sausage mix. The discrepancy between both studies could be attributed to differences in the antimicrobial source used, the making process, and storage conditions (i.e., 50 °C smoking, 20–22 °C fermentation and 10–12 °C ripening-drying steps versus continuous refrigerated storage under CO_2_-containing MAP).

Storage time affected the amounts of all mono and diamines in the fresh sausage, which increased steadily, indicating a continuous formation of those BA by the active microbiota during storage. However, time did not affect the content of polyamines originated from de novo synthesis in animal tissues [44]. Overall, the time-related changes in the content of BA in this study have been observed in other studies on fresh sausages stored under anaerobic MAP [2,8]. Biogenic monoamines can produce toxic effects on the consumers resulting in migraine, hypertensive crisis, or allergy [45]. Among them, histamine and tyramine present the highest health concern [46]. The maximum recommended levels for both amines in fermented sausages are over 100 mg/kg—although their toxicity depends not only on their levels in food but also on dietary factors and consumers’ susceptibility [47]. Tyramine content in the fresh sausages from this study exceeded that limit (100 mg/kg) at day 14. On the other hand, although the diamines putrescine and cadaverine are not considered toxic per se, they can enhance the toxic effect of histamine and tyramine [48].

### 2.2. Identification of Lactic Acid Bacteria

Only eight out of the 346 isolates from the DeMan-Rogosa-Sharpe (MRS) agar plates were not positively identified. Among the identified isolates, 90% corresponded to LAB (70% of isolates were identified as LAB at day 0 and ≥90% at the other sampling days). Among the non-LAB bacteria, the genus identified in order of abundance were *Staphylococcus* spp., *Enterobacter* spp., *Serratia* spp., *Filifactor* spp., *Escherichia* spp., and *Macrococcus* spp. (not shown in tables). Table 2 shows the frequency (%) of the LAB species identified at different storage days considering the isolates in sausages from the four antimicrobial treatments. The genus *Lactobacillus* was the most abundant (84% of the LAB isolates) regardless of the storage time. *Lactobacillus sakei* was the predominant LAB, with its frequency overcoming 50% from day 7 onwards—when LAB counts showed significant growth (counts higher than 7 Log CFU/g; Figure 1). Among the isolates identified as *Lb. sakei*, 59% were identified as *Lb. sakei* subsp. *carnosus* (not shown in tables) and the others were identified as *Lb. sakei* (only species level). On the other hand, approximately 40% of the isolates ascribed to the *Lactobacillus* genus were not positively identified at species level (provided as *Lactobacillus* spp. in Table 2). Comparing between the first weeks (especially day 0) and the last weeks of storage, the diversity (number) of LAB species showed a tendency to decrease. On days 28 and 35, there was a clear dominance of *Lactobacillus*, and among them, *Lb. sakei* (<90% and ≤70% of total LAB, respectively).

**Figure 2:**
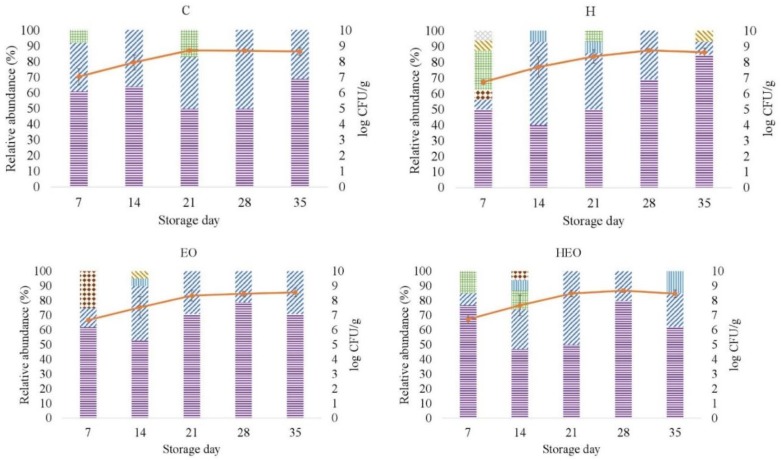
Relative abundance (%) and growth curve (mean and standard deviation, vertical bars; *n* = 3) of the isolates identified as lactic acid bacteria in fresh lamb sausages packaged under modified atmosphere (80% N_2_, 20 %CO_2_) during refrigerated storage (4 °C). CFU: Colony forming unit. C: Control sausages; H: hops; EO essential oil (*Zataria multiflora* Boiss); HEO: hops and essential oil. *Lactobacillus sakei* (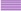), *Lactobacillus* spp. (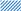), *Leuconostoc mesenteroides* (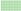), *Enterococcus faecalis* (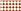), *Carnobacterium maltromaticum* (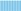), *Lactobacillus curvatus* (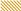), *Lactococcus lactis* (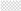).

Figure 2 depicts the frequency of LAB species or genus, as identified by the matrix-assisted laser desorption/ionization-time of flight (MALDI-TOF) analysis, obtained for each of the experimental treatments from day 7 to 35. *Lb. sakei* was predominant for each of the treatment-day combinations except for H-day 14 (ranging from 40% to 85%). The contingency chi-square test showed no significant effect of treatment either on the frequency of *Lb. sakei* or on *Lactobacillus* spp. (*P* = 0.419 and *P* = 0.729, respectively).

Most of the studies on the succession of microbial population in fresh sausages during refrigerated storage have been carried out with the sausages stored under aerobic normal or modified atmosphere [1,4,6,32]. In these studies, LAB species have been one of the major microorganisms detected together with species belonging to *Enterobacteriaceae*, *Micrococcaceae*, *Pseudomonas* spp., and *B. thermosphacta*. However, LAB (*Lb. sakei*, *Lb. curvatus*/*graminis*) and *B. thermosphacta* tended to be the most predominant groups at the end of storage. The degree of abundance of LAB would directly depend not only on storage time but also on the reduction degree of redox potential during storage [49]. On the other hand, in the study by Fougy et al. [7], the sausages were packaged under vacuum and anaerobic 50% CO_2_-containing atmosphere. The authors, in agreement with the results of the present study, found, using metagenetic 16S rRNA pyrosequencing, *Lb. sakei* to be the most abundant species in spoiled sausages (after 21 days of storage). Moreover, they also reported the presence of *Lactococcus piscium*, *Carnobacterium divergens*, *Carnobacterium maltaromaticum*, *Serratia proteamaculans*, and *B. thermosphacta*.

*Lb. sakei* together with *Lb. curvatus* appears to be the dominant LAB species in fermented sausages produced by spontaneous fermentation, and *Lb. sakei* is the most used LAB species as a starter culture for these sausages [50]. This species has been demonstrated to have an effective metabolic adaptation to the meat environment, and to cold temperature and high NaCl concentration. These abilities explain its growth in refrigerator-stored fresh sausages. This LAB have been demonstrated to be resistant to the presence of the antimicrobials tested in the present study at the levels used.

In contrast with traditional fermented sausages, fresh sausages became spoiled after fermentation, which would be due to differences in the LAB metabolism in a meat environment with higher a_w_ and the concomitant activity of other spoilage microorganisms, i.e., *B. thermosphacta* and *Enterobacteriaceae*.

## 3. Materials and Methods

### 3.1. Experimental Plan

Three batches of ćevapi, a Bosnian-style fresh sausage, were prepared using lamb lean meat at the pilot plant at the Faculty of Veterinary Medicine, University of León (Spain). Each batch was composed of four treatments: control (C), with no antimicrobial additives in the formulation; hops extract (H), including an aqueous hops extract; essential oil (EO), with ZM’s EO, and with both hops extract and essential oil (HEO). Sausages were packaged in bags under a modified atmosphere (20% of CO_2_ and 80% N_2_) and then stored under refrigeration at 2 °C for 35 days. Water activity (a_w_) was determined at the day of packaging (day 0), and pH, the presence of relevant microbial groups, i.e., lactic acid bacteria (LAB), *Enterobacteriaceae, Brochothrix thermosphacta, Micrococcaceae* and yeast and mold, and the concentration BA were analyzed weekly (storage days 0, 7, 14, 21, 28, and 35). Moreover, 346 colonies (4–5 per batch, day, and treatment) were picked from De Man Rogosa agar plates used for LAB counts and identified by MALDI-TOF mass spectrometry.

### 3.2. Lamb Meat, Hops Extract, and Essential Oil

Lamb meat came from the legs of six male Assaf lambs reared at the Instituto de Ganadería de Montaña (CSIC; Grulleros, León, Spain). The lambs were weaned with 14 ± 2 kg of weight and fattened to 50 ± 4 kg of weight ad libitum on a pelleted complete diet based on straw (150 g/kg), cereals (barley, corn and soybean meal; 810 g/kg), molasses (10 g/kg), a mineral-vitamin premix (25 g/kg), and sodium bicarbonate (5 g/kg). The animals were then slaughtered in a local abattoir, and their legs were separated from the right-hand carcasses after 24 h post-mortem and then deboned. The meat was then cut into approximately 3 cm cubes, which were trimmed of visible fat. The lean meat from each leg was packaged under vacuum and frozen (−20 °C) until being used (up to 3 months).

The aqueous hops extract used in the study was obtained from recently cropped Nugget variety hop, with *α*-acid, *β*-acid, and co-humulone composition of 4.8–5.3%, 12–16%, and 22–28%, respectively. The hops was provided by a local producer (Orbigo Valley S.L., Madrid, Spain). An amount of 50 g of hops was boiled into 1 L of water for 30 min and the final volume was filled up to 1 L, which was filtered through a Whatman number 1 filter paper (GE Healthcare Europe, Barcelona, Spain) and frozen at −18 °C until further use. ZM’s EO was obtained from the Faculty of Agriculture, University of Tehran, Iran. Crushed dried leaves of ZM plant were transferred to an all-glass Clevenger-type apparatus and steam distilled for 2.5 h. The essential oil was then dried over anhydrous Na_2_SO_4_ and stored in opaque glass bottles until further use.

### 3.3. Sausage Manufacture

Three batches of sausages were produced on different days using the meat from the legs of 2 among the 6 lambs for each of the batches. The sausage-making process was based on a Balkans-style ćevapi recipe. Lamb meat was thawed at 5 °C for 24 h and minced using a butcher’s mincer equipped with a 5 mm diameter sieve. A total of 3.8 kg of minced meat was mixed with salt (80 g) for 10 min and placed into a bowl covered with cling film and stored at 4 °C until the next day (24 h). A mixture of finely cut fresh garlic and pepper was boiled in water for 2 min. The mixture (spices solution) was cooled, filtered, and then stored (4 °C) until the next day. The salted minced meat was divided into four parts of 950 g each, one for each of the four above-mentioned treatments (C, H, EO, and HEO) using the ingredients provided in Table 3. All the portions were mixed (for 5 min) with the spice solution (20 mL/kg) and 3 g/kg of sodium bicarbonate. C, EO, and HEO were also mixed with an amount of water, H and HEO treatment with hops extract, and EO and HEO with essential oil (see Table 3). The amount of hops extract added to the H sausage was equivalent to 1.5 g of hops per kg of sausage (i.e., 30 mL of the solution obtained from boiling 50 g of hops per L), which is that commonly used in brewery. The amount of ZM’s EO used (1 mL/kg) was within the concentration ranges reported for antimicrobial activity of EOs in food [16], i.e., around 0.5–20 mL of EO per kg. When both antimicrobial sources were added, their amounts were halved.

The sausage mixtures were stuffed into lamb casings (20/22 cm diameter) and drained for 3 h at 12 °C. The sausages were then cut into 100 g portions, which were individually packaged in bags (150 μm plastic film, oxygen permeability of 30 cm^3^/(m^2^ × bar × 24 h) at 23 °C and 0% relative humidity) under a 20% CO_2_ and 80% N_2_ atmosphere at 750 mbars, and refrigerator-stored (2 °C). One C portion was used for analysis at day 0 and one packaged portion for each of the treatments was sampled after 7, 14, 21, 28, and 35 days of storage for subsequent analysis.

### 3.4. Analysis of Water Activity, pH, Microbial Content, and Biogenic Amine Production

Water activity (a_w_) was determined in duplicate at 25 °C using a CX-2 hygrometer (Decagon Devices Inc., Pullman, WA, USA) following the manufacturer’s instructions, and pH using a pHmeter (Model 507; Crison, Barcelona, Spain) according to the International Organization for Standardization (ISO) guideline 2917 [51]. For microbiological analysis, samples of 25 ± 0.1 g of sausages were homogenized with 225 mL of peptone water (0.1% peptone) for 2 min in sterile bags using a Stomacher-400 circulator (Seward, West Sussex, UK). Serial decimal dilutions were prepared, and aliquots of the appropriate dilutions were cultured in duplicate on the corresponding media and incubated, according to the procedure described by the culture media manufacturer, as follows: 1 mL on the De Man-Rogosa-Sharpe agar (Oxoid) with double agar layer at 30 °C for 72 h for LAB; 1 mL in Mannitol Salt Agar (Oxoid) at 35 °C for 48 h for *Micrococcaceae*; 1 mL in Violet Red Bile Glucose Agar (VRBGA; Oxoid) with double agar layer at 35 °C for 48 h for *Enterobacteriaceae*; 1 mL in Oxytetracycline Glucose Yeast extract agar (Oxoid) at 22 °C for 5 days for molds and yeast, and 0.1 mL onto the surface of STAA Agar Base (CM 0881; Oxoid) plates containing STA Selective Supplement (0.4 mL/100 mL) and sterilized glycerol (1.5 g/100 mL), at 22 °C for 48 h (only the straw colored oxidase-negative colonies were considered).

Biogenic amine contents were analyzed following the Eerola, Hinkkanen, Lindfors, and Hirvi [52] procedure using a high performance liquid chromatograph (HPLC) Alliance (Waters 2695) equipped with a double wavelength detector (Waters 2996, Waters Corporation, Milford, MA, USA) and a Spherisorb ODS2 column (125 × 4 mm ID; 5 μm; Waters). The standards used for detection and quantification were tryptamine cadaverine dihydrochloride, histamine dihydrochloride, putrescine dihydrochloride, spermidine, spermine, tryptamine hydrochloride, and tyramine hydrochloride (Sigma-Aldrich Química, Madrid, Spain).

### 3.5. Identification of Lactic Acid Bacteria

From the growth in the MRS plates, 4–5 colonies were picked for each experimental treatment (4), sampling day (7) and batch (3), giving 346 colonies in total. These isolates were then grown in Tryptone Soy Broth (TSB; Bacto, Mt Printchard, Australia) with 0.5% (*w*/*v*) of yeast extract (YE; Difco, Leeuwarden, The Netherlands) (TSB-YE) at 37 °C for 24 h. One mL aliquot was centrifuged (12,000 rpm, 3 min) in Eppendorf tubes (Eppendorf Ibérica, San Sebastián de los Reyes, Madrid). The supernatants were discarded, and the pellets were suspended in 1 mL of MRS broth with 50% (*v*/*v*) of glycerol. The isolates were maintained at −40 °C for storage purposes. Isolates were recovered for their identification as follows: they were grown at 30 °C on MRS broth (Oxoid) with 0.5% (*w*/*v*) of YE (Difco) at 37 °C for 24 h, and then a loopful of bacteria was sub-cultured in MRS agar (Oxoid).

The analysis was carried out at the Laboratory for Instrumental Analysis, University of Valladolid (Valladolid, Spain). For the analysis, one colony from the MRS plate was picked using a sterilized toothpick and smeared gently onto a MALDI-TOF target plate (Bruker Daltonik GmbH, Leipzig, Germany). After air-drying, 1 µL of formic acid was added. The dried sample was overlaid with 1 μL matrix solution containing 10 mg/mL *α*-cyano-4-hydroxycinnamic acid (HCCA) in a mixture of acetonitrile, deionized water, and trifluoracetic acid (50/47.5/2.5, *v*/*v*/*v*). The target plate with samples were introduce in the MALDI-TOF equipment for analysis. Not all the bacteria were amenable to analysis: approximately 30% of the isolates were not accurately identified, i.e., identification score at genus level <1.7. For these isolates, the analysis was repeated including an ethanol extraction tube protocol before analysis to extract ribosomal proteins according to the manufacturer’s instructions (Bruker Daltonik). Briefly, one colony from MRS plates was sub-cultured in TSB + 0.5% (*w*/*v*) of yeast extract at 35 °C overnight. One mL aliquot of the isolate was transferred into an Eppendorf tube and centrifuged at 12,000 rpm for 2 min. The supernatant was discarded, and the pellet was mixed thoroughly with 1 mL of deionized water and centrifuged at the same speed and time. This stage was performed twice. Afterward, 900 µL of absolute ethanol and 300 µL deionized water were added, mixed for 2 min, and the tube was centrifuged at 15,000 rpm for 5 min. The supernatant was discarded, and the pellet was air-dried for a minimum of 30 min until dryness. The pellet was re-suspended with 15 µL of formic acid (70%) and mixed thoroughly. Moreover, the mix was kept for 5 min at room temperature and then 15 µL of acetonitrile was added and mixed. The mixture was centrifuged at 15,000 rpm for 3 min and subsequently, 1 mL of the supernatant was spotted onto a MALDI-TOF target plate. After being air-dried, the sample was overlaid with 1 μL of matrix solution (HCCA).

For identification, each series of measurements was preceded by a calibration step with a bacterial test standard (BTS 155 255343; Bruker Daltonik) to validate the run. Mass spectra were generated by a Flex Analysis MALDI-TOF mass spectrometer (Bruker Daltonik) equipped with a nitrogen laser (l1⁄4337 nm) operating in linear positive ion detection mode under the Bruker Flex Control software (Bruker Daltonik). The Autoflex LT Speed was periodically calibrated by using the Bruker Daltonik *Escherichia coli* bacterial test standard DH5. Automated analysis of the raw spectral data was performed by the MALDI BioTyper automation (version 3.1) software (Bruker Daltonik) using a library of 5627 main spectra (MSPs; database update of 7/15/2015). Identifications at species or genus level were considered if scores were above 2.0 and 1.7 respectively, according to the report generated by Bruker Compass [53,54].

### 3.6. Statistical Analysis

Data on microbial counts and BA levels were analyzed by two-way analysis of variance (ANOVA) with treatment and storage day as fixed factors. When the fixed factors or their interaction showed significant differences (*P* < 0.05), the ANOVA was followed by the Tukey’s post-hoc test. For the results of the LAB identification, a contingency table (4 by 2; treatment by positive or negative) chi-square analysis was used to test the eventual dependence between treatment and the frequency of the presence in the sausages of the main genus or species identified, considering the entire storage period. The statistical analysis was performed using the SPSS Statistics software (version 24; IBM, Somers, NY, USA).

## 4. Conclusions

The results from this study demonstrate that Balkan-style fresh sausages stored under anaerobic atmosphere are already fermented in the first week of storage and the predominant responsible species are *Lactobacillus* spp., specifically *Lb. sakei*. The fermentation was compatible with a controlled growth of *B. thermosphacta* and *Enterobacteriaceae* and resulted in BA production to a concerning level, thus suggesting that the contents of BA in anaerobic MAP fresh sausages should be controlled. The use of *Zataria multiflora* Boiss EO, hops extract, or the combination of both at the levels used did not significantly affect the microbial development in the sausages. More studies using higher amounts of these antimicrobial sources, different combinations with other antimicrobials, extracts with higher concentrations of active compounds, or previous encapsulation, would be needed to achieve their effectiveness in fresh sausage preservation.

## Figures and Tables

**Table 1:** Biogenic amine contents (mg/kg) in fresh sausages stored at 2 °C under anaerobic modified atmosphere storage as a function of antimicrobial treatment (Treat) and storage day (Time).

Biogenic Amine	Treat				Time						RMSE	*P*-Value		
	C	H	EO	HEO	0	7	14	21	28	35		Treat	Time	Treat × Time
Monoamines														
Tryptamine	17.66	20.48	17.52	19.08	5.58 ^b^	6.28^b^	16.85 ^ab^	18.80 ^ab^	29.61 ^a^	34.99 ^a^	16.33	0.942	0.000	1.000
Histamine	6.90	7.58	4.53	8.24	0.53 ^b^	0.37 ^b^	2.40 ^b^	4.31 ^b^	13.96 ^ab^	19.29 ^a^	17.48	0.926	0.046	1.000
Tyramine	143.04	152.28	150.89	150.40	19.88 ^e^	99.04 ^d^	138.37 ^cd^	176.22 ^bc^	206.08 ^ab^	255.31 ^a^	44.25	0.924	0.000	1.000
Diamines														
Putrescine	8.36	6.97	6.04	6.99	1.36 ^b^	1.86 ^b^	3.06 ^ab^	7.03 ^ab^	12.23 ^ab^	17.00 ^a^	12.35	0.955	0.015	1.000
Cadaverine	98.03	121.74	126.37	114.29	2.14 ^c^	14.33 ^c^	66.69 ^c^	148.73 ^b^	203.61 ^ab^	255.14 ^a^	54.56	0.432	0.000	0.996
Poliamines														
Spermine	25.52 ^b^	28.88 ^ab^	29.22 ^ab^	29.89 ^a^	30.12	30.17	26.57	26.88	27.16	29.34	4.72	0.037	0.200	0.506
Spermidine	5.83	5.80	6.06	6.22	5.56	6.02	5.97	6.06	5.87	6.37	1.24	0.715	0.738	0.587

C: Control sausages; H: hops; EO essential oil (*Zataria multiflora* Boiss); HEO: hops and essential oil. ^a-e^: Means in the same row within treatment or time showing different superscripts are significantly different (*P* < 0.05; Tukey test).

**Table 2:** Lactic acid bacteria (LAB) species in the sausages ^#^ at the different storage days (expressed in % of total isolates identified as LAB).

Species	0 (*n* = 33)	7 (*n* = 50)	14 (*n* = 63)	21 (*n* = 52)	28 (*n* = 56)	35 (*n* = 53)
*Aerococcus viridans*	12	-	-	-	-	-
*Carnobacterium maltraromaticum*	-	-	5	2	-	4
*Lactobacillus casei*	3	-	-	-	-	-
*Lactobacillus curvatus*	-	2	2	-	-	2
*Lactobacillus sakei*	33	62	51	56	70	72
*Lactobacillus* spp.	12	14	38	37	30	23
*Lactococcus lactis*	24	2	-	-	-	-
*Leuconostoc mesenteroides*	12	14	3	6	-	-
*Enterococcus faecalis*	-	6	2	-	-	-
*Streptococcus salivarius*	3	-	-	-	-	-

*n* = number of isolates identified as lactic acid bacteria; ^#^ Results on each day include the isolates from the four antimicrobial treatments.

**Table 3:** Ingredients and amounts (expressed in g or mL, solid or liquids, respectively) used in the sausage preparation for the experimental treatments.

Ingredients	Treatments			
	C	H	EO	HEO
Lamb meat	980	980	980	980
Salt	20	20	20	20
Sodium bicarbonate	3	3	3	3
Spice infusion ^a^	20	20	20	20
Water	30	-	30	15
Hops extract	-	30	-	15
Essential oil	-	-	1	0.5

C: Control sausages; H: hops; EO essential oil (*Zataria multiflora* Boiss); HEO: hops and essential oil. ^a^: Filtered solution obtained by boiling garlic and pepper in water.
